# Oxidative Stress and TGF-*β*1/Smads Signaling Are Involved in* Rosa roxburghii* Fruit Extract Alleviating Renal Fibrosis

**DOI:** 10.1155/2019/4946580

**Published:** 2019-08-20

**Authors:** Jihong Zhan, Mingjie Liu, Lijun Pan, Liqun He, Yinxue Guo

**Affiliations:** ^1^Department of Nephrology, The First Affiliated Hospital of Guiyang Traditional Chinese Medicine College, Guizhou, China; ^2^Department of Nephrology, Shanghai Shuguang Hospital Affiliated to Traditional Chinese Medicine, Shanghai, China

## Abstract

Fibrosis is involved in the pathogenesis of kidney diseases. We previously discovered that* Rosa roxburghii* fruit (Cili) possesses antifibrosis property in chronic renal disease, but the mechanisms are unknown. We hypothesized that Cili might prevent fibrosis development through mediating TGF-*β*/Smads signaling, which is known to be involved in renal fibrosis. This study aimed to confirm the effects of freeze-dried Cili powder in a rat model of unilateral ureteral obstruction (UUO) and examine TGF-*β*/Smads signaling. Rats were randomized to (n=12/group): sham operation, UUO, UUO with losartan, UUO with moderate Cili dose (3 g/kg/d), and UUO with high Cili dose (6 g/kg/d). The rats were sacrificed after 14 days of treatment. Collagen deposition was tested using Masson's staining. TGF-*β*/Smads signaling was examined by qRT-PCR, western blot, and immunohistochemistry. Rats in the UUO group showed excessive deposition of collagen in kidney interstitium, accompanied with high levels of renal 8-hydroxy-2′-deoxyguanosine, renal malondialdehyde, blood urea nitrogen (BUN), serum creatinine (Scr), and proteinuria (all P<0.05). Cili powder efficiently alleviated the pathological changes and oxidative stress in the kidneys of UUO rats, and decreased BUN, Scr and proteinuria (all P<0.05). Cili powder also inhibited the upregulation of TGFB1, TGFBR1, TGFBR2, SMAD2, and SMAD3 and reversed the downregulation of SMAD7 in obstructed kidneys (mRNA and protein) (all P<0.05). In summary, the results suggest that Cili freeze-dried powder effectively prevents renal fibrosis and impairment in UUO rats, which is associated with the inhibition of oxidative stress and TGF-*β*1/Smads signaling.

## 1. Introduction

Renal fibrosis is a consequence of progressive kidney diseases such as chronic infection, obstruction of the ureters, hypertension, and diabetic nephropathy [1, 2]. Renal fibrosis is characterized by tubulointerstitial fibrosis and glomerulosclerosis, both caused by the excessive deposition of collagens and extracellular matrix (ECM) components. Although the kidney can regenerate after acute injury, renal fibrosis is usually irreversible [3].

Transforming growth factor-beta (TGF-*β*)/Smads signaling plays a pivotal role in renal fibrosis [4–6]. Elevated TGF*β*1 and downstream Smad3 and Smad2 in the kidney activate profibrotic genes and mediate renal fibrosis [5]. On the other hand, Smad7 suppresses renal fibrosis by altering the expression of TGF-*β*/Smad3 through microRNAs [7]. Inhibition of the TGF-*β*1/Smad2/3 pathway alleviates kidney injury and renal fibrosis [8, 9].

The traditional Chinese medicine (TCM) is based on a personalized approach aiming to restore the balance between Yin and Yang [10, 11]. There are hundreds of TCM formulation against chronic kidney diseases (CKD) [12, 13]. Some TCM components were studied (including* Astragalus*,* Angelica sinensis*,* Rheum plantarum*,* Radix bupleuri*, Cordyceps sinensis, and* Tripterygium wilfordii *[11]) and their effects include anti-inflammation, antioxidation, antifibrosis, anticoagulation, and immune system modulation [13, 14]. On the other hand, some TCM components can be toxic to the kidney [11, 15], hence the importance of characterizing them carefully.

*Rosa roxburghii* is a member of the rose family and is a wild plant from the Guizhou Province and distributed in south China. In TCM, the fruit of* Rosa roxburghii* (named Cili or CL) improves immune response, enhances digestive ability, and possesses antiaging effects [16]. Chemical analysis revealed that Cili is rich in antioxidants including superoxide dismutase (SOD), vitamin C, vitamin E, and flavonoids [17, 18]. Cili has a long history in China and has been proven to possess antioxidant [19, 20], antiatherosclerosis [21], antitumor [22], and radioprotective [23] effects. Nevertheless, the exact molecular mechanisms responsible for these beneficial effects are not well understood.

Our preliminary data showed that freeze-dried Cili powder improved renal fibrosis and indexes of oxidative stress in 90 patients with stages 3-4 renal failure [24], but the exact mechanisms responsible for these effects of Cili on kidney diseases are unknown. We hypothesized that Cili could prevent the development of renal fibrosis through mediating TGF-*β*/Smads signaling, which is known to be involved in renal fibrosis [4–6]. Therefore, this study established a renal fibrosis rat model induced by unilateral ureteral obstruction (UUO) in order to confirm the effects of freeze-dried Cili powder on renal fibrosis and examine TGF-*β*/Smads signaling.

## 2. Materials and Methods

### 2.1. Animals

Sixty specific pathogen-free- (SPF-) grade male Sprague-Dawley rats (140-160 g) were provided by Shanghai Sippr/BK Laboratory Animal Ltd., Co. (Shanghai, China; certificate number: DA942C48-72F76A1E-466ABC83-FC6974). The rats were kept at the Experimental Center for Science and Technology, Shanghai University of Traditional Chinese Medicine, under controlled temperature (25°C), humidity (45-55%), and light (12 h light/dark cycle). The animals were allowed free access to food (standard chow) and water. All rats were kept one week before experiments. All experimental procedures were performed according to the local Animal Care Committee's guidelines.

### 2.2. Unilateral Ureteral Obstruction (UUO) Modeling and Treatment

According to the principles of sample size estimation in Chinese medicine pharmacology research [25], “When using small animals (mice or rats) to carry out the experiments and comparing categorical data among 3-5 groups, the number of animals in each group should be no smaller than 8-10”. Some other studies with a similar methodology also used 8-10 rats/group [26, 27]. Being prudent, a sample size of 12 rats/group was used in the present study. The rats were randomized to (n=12/group): sham operation group (Sham), UUO group (UUO), UUO rats treated with losartan (UUO+losartan), UUO rats treated with moderate dose of Cili freeze-dried powder (UUO+CL moderate), and UUO rats that received high dose of Cili freeze-dried powder (UUO+CL high). Rats that underwent UUO and were treated with resveratrol were used as a positive control group.

UUO was performed as previously described [28]. Briefly, animals were anesthetized with 2% pelltobarbitalum natricum (0.6-0.8 mL/rat). The left kidney was exposed and separated through a 2-3 cm flank incision. The left ureter was ligated at two locations with 4-0 silk and cut between the two ligatures. In the sham-operated rats, the left ureter was separated but not ligated or cut.

All rats were treated on the next day after successful modeling. The conversion formula for the drug dose in rats (in g/kg) is [clinical dose in humans, in g/kg]×6.2 [29]. Our previous study found that the equivalent dose for patients with chronic renal failure at stages 3-4 was about 40 g/day of freeze-dried powder of* Rosa roxburghii* fruit (Pharmacy Department, The First Affiliated Hospital, Guiyang College of Traditional Chinese Medicine) by oral administration [24]. The reference human body weight is about 60 kg [29]. Therefore, the equivalent dose for rats using this kind of Cili freeze-dried powder should be 40 g/60 kg × 6.2 =4 g/kg. The content of active ingredients (flavonoids) is twice higher in commercially available Cili freeze-dried powder (GuizhouJincili Technology Development Co., Ltd., Guizhou, China) compared with home-made preparation, due to optimized manufacturing processes [30]. Hence, the equivalent dose for rats using the commercially available Cili freeze-dried powder is 2 g/kg. In the present study, the dose of Cili freeze-dried powder (Guizhou Jincili Technology Development Co., Ltd., Guizhou, China) was 3 g/kg/d for the UUO+CL moderate group (1.5 times the equivalent dose) and 6 g/kg/d for the UUO+CL high group (three times the equivalent dose). The rats in the sham and UUO groups were given the same volume of sterile saline. Losartan (losartan potassium, lot: L037309; Merck Frost, Montreal, Canada) was given by oral gavage at 10 mg/kg/d (20% concentration). All experimental procedures and care were performed at the same time, once per day, for 14 days. According to previous studies [31, 32], early renal fibrosis is observed in UUO rats from 3 days after surgery, most of the quantitative pathological changes occur about 7 days after surgery, and obvious renal fibrosis is observed on the 14th day after surgery. Therefore, 14 days of treatment was used for the prevention of renal fibrosis in the present study. According to similar studies [33, 34], 14 days of traditional Chinese medicine intervention in UUO model rats showed treatment effectiveness.

The rats were sacrificed on day 14 after gavage under general anesthesia with 3.5% isoflurane. Both kidneys were collected, rinsed with ice-cold saline, dissected and frozen in liquid nitrogen or fixed in 5% PBS-buffered formalin, rinsed with phosphate buffer, dehydrated using a graded series of ethanol and xylene, and embedded in paraffin.

### 2.3. Histopathology

Paraffin sections (4 *μ*m thick) were stained with hematoxylin and eosin (HE) for histopathological examination. Kidney fibrosis was evaluated by Masson's trichrome staining, as described previously [35]. The amount of collagen-positive (blue) pixels in the stained sections was quantified by automatic analysis using the Image-Pro Plus 6 software (Media Cybernetics, Rockville, MD, USA). Eight random fields on each section were selected for analysis. The area of interest (AOI) was acquired based on the total amount of stained tissue per high-power field. Integrated optical density (IOD) of blue pixels was determined and the percentage of blue pixels in the AOI was automatically calculated.

### 2.4. Immunohistochemistry

Paraffin-embedded kidneys were sectioned at 4 *μ*m. The sections were air-dried, dewaxed, and rehydrated in graded ethanol and phosphate buffered saline (PBS). Endogenous peroxidase activity was blocked using 3% hydrogen peroxide and the sections were blocked with 10% goat serum. The primary antibodies were anti-TGF-*β*1(1:200; Boster Bioengineering Co., Wuhan, China), anti-TGF-*β*1 (1:200; Santa Cruz Biotechnology, Santa Cruz, CA, USA), anti-Smad2 (1:200; #ab40855; Abcam, Cambridge, MA, USA), anti-Smad7 (1:200; #BA1499, Boster Bioengineering Co., Wuhan, China), and anti-Smad3 (1:1000; #ab40854, Abcam, Cambridge, MA, USA). All sections were subsequently incubated with the respective secondary antibodies at room temperature for 1 h. Peroxidase-labeled polymer and diaminobenzidine (DAB) were used to visualize staining. Three to five sections of each specimen were used for immunohistochemistry. A qualitative analysis was first performed; according to the location of the specific protein in the control group (e.g., the cortical part), the same location was analyzed in the treatment groups. Semiquantitative analysis was then conducted using eight randomly selected nonoverlapping fields (×400) from each section. The quantitative analysis was conducted using Image Pro Plus 6.0.

### 2.5. Measurement of Oxidative Stress Markers

Rat kidneys were minced and added to normal saline [for 8-hydroxy-2′-deoxyguanosine (8-OHdG) measurement, the solution was PBS (pH 7.4) instead] using a ratio of weight (g):volume (ml) of 1:9. The samples were homogenized on ice and centrifuged at 2750 rpm for 10 min, and the supernatant was collected. The protein concentration was determined using the Coomassie bright blue kit (Jiancheng Bioengineering Institute, Nanjing, Jiangsu, China). SOD activity was determined using the xanthine oxidase method. The samples were incubated with the substrate, enzyme, and buffer mixture at 37°C for 20 min, according to the manufacturer's recommendations (Jiancheng Bioengineering Institute, Nanjing, Jiangsu, China). The reaction product was detected at 450 nm. The results were expressed as units per milligram of protein (U/mg protein). MDA amount was determined using the thiobarbituric acid (TBA) method. The samples were incubated with the reaction buffer at 95°C for 40 min, according to the manufacturer's recommendations (Jiancheng Bioengineering Institute, Nanjing, Jiangsu, China). The MDA-TBA product was detected at 535 nm. The results were expressed as nmol per milligram of protein (nmol/mg protein). 8-OHdG amount was determined using competitive ELISA (No. H165; Jiancheng Bioengineering Institute, Nanjing, Jiangsu, China).The microplates were read at 450 nm.

### 2.6. Quantitative Real-Time PCR

Total RNA was isolated from frozen kidney samples and subjected to reverse transcription to obtain cDNA. Quantitative real-time PCR was performed using the SYBR Green PCR Kit (Qiagen, Venlo, The Netherlands) on a LightCycler® 480 System (Roche Molecular Systems, Pleasanton, CA, USA). GAPDH was used as internal reference. The RT-PCR primers are listed in Table 1. The PCR conditions were (1) 95°C for 30 s and (2) 40 cycles of 95°C for 20 s, 60°C for 20 s, and 72°C for 20 s.

### 2.7. Western Blot

Western blot analyses were performed using antibodies against TGF-*β*R1 (1:50; Wuhan Boster Bio-Engineering Limited Company, Wuhan, China), TGF-*β*R2 (1:50; Wuhan Boster Bio-Engineering Limited Company, Wuhan, China), Smad7 (1:200; #BA1399; Wuhan Boster Bio-Engineering Limited Company, Wuhan, China), SIRT1 (1:200; #ab110304; Abcam, Cambridge, MA, USA), Smad2 (1:200; #ab40855; Abcam, Cambridge, MA, USA), Smad3 (1:200; #ab40854; Abcam, Cambridge, MA, USA), TGF-*β*1 (1:500; #ab92486; Abcam, Cambridge, MA, USA), and GAPDH (1:10,000; Abcam, Cambridge, MA, USA).

### 2.8. Biochemistry

The serum levels of blood urea nitrogen (BUN) and creatinine (Scr) were detected with assay kits from the Nanjing Jiancheng Bioengineering Institute (C013-1 for BNU and C011-1 for creatinine; Nanjing, China), according to the manufacturer's instructions. Twenty-four-hour urine samples were collected using stainless steel metabolic cages at indicated time points. Urine was centrifuged at 12,000 ×g for 15 min at 4°C and the supernatant was used for detection. Urine protein (Upro) concentrations were determined using a urinary protein assay kit (C035-2; Nanjing Jiancheng Bioengineering Institute; Nanjing, China).

### 2.9. Statistical Analyses

All data were verified using the Kolmogorov-Smirnov test and were found to follow the normal distribution. The results were expressed as means ± standard deviation. The differences among the groups were analyzed using one-way analysis of variance (ANOVA) followed by the least significant difference (LSD) post hoc test. All analyses were performed using SPSS 18.0 (IBM, Armonk, NY, USA). The differences were considered statistically significant at P<0.05 (two-sided).

## 3. Results

### 3.1. Analysis of the Freeze-Dried Powder of* Rosa roxburghii*

In order to ensure the consistency among batches of freeze-dried powder of* Rosa roxburghii*, samples were sent to and tested by the China National Analytical Center in Guangzhou (China). The results showed that the flavonoids content was the same (0.4%) for all three batches, while the polysaccharides contents were 5.5%, 6.2%, and 5.7%, respectively (mean of 5.8%).  Table 2 presents the exact content of some nutrients. SOD activity was 2.88 × 10^4^ U/g.

### 3.2. Cili Administration Inhibits the Pathological Changes in Kidney after UUO

**Figure 1:**
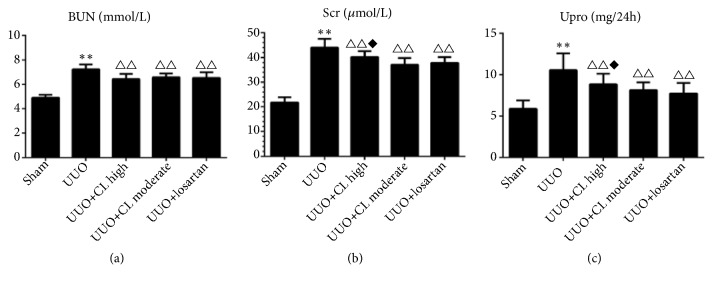
*Cili alleviates the functional changes in kidneys after unilateral ureteral obstruction (UUO)*. Sixty rats were randomized to five groups: sham operation, UUO, UUO with losartan (UUO+losartan), UUO with moderate Cili dose (3 g/kg/d) (UUO+CL moderate), and UUO with high Cili dose (6 g/kg/d) (UUO+CL high). Biochemical markers indicative of kidney functions were measured, including (a) blood urea nitrogen (BUN), (b) serum creatinine (Scr), and (c) urine protein (Upro) levels. N=12/group. The results are expressed as mean ± standard deviation (SD). *∗∗*P<0.01 vs. the sham group; △△P<0.01 vs. the UUO group; ◆P<0.05 vs. the losartan group.

**Figure 2:**
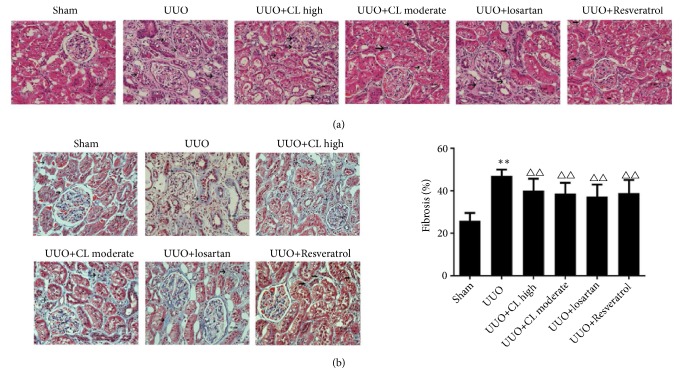
*Cili alleviates the morphological changes in kidneys after unilateral ureteral obstruction (UUO)*. Sixty rats were randomized to six groups: sham operation, UUO, UUO with losartan (UUO+losartan), UUO with moderate Cili dose (3 g/kg/d) (UUO+CL moderate), UUO with high Cili dose (6 g/kg/d) (UUO+CL high), and UUO with resveratrol (UUO+Resveratrol). (a) Hematoxylin and eosin (H&E) staining of kidneys was performed. Lesions such as glomerular hypertrophy and vacuolation of renal tubular epithelial cells are indicated by arrows. (b) Masson's staining of kidneys and quantification of the fibrosis area. Scale bar: 10 *μ*m. N=12/group. The results are expressed as mean ± standard deviation (SD). *∗∗*P<0.01 vs. the sham group; △△P<0.01 vs. the UUO group.

**Figure 3:**
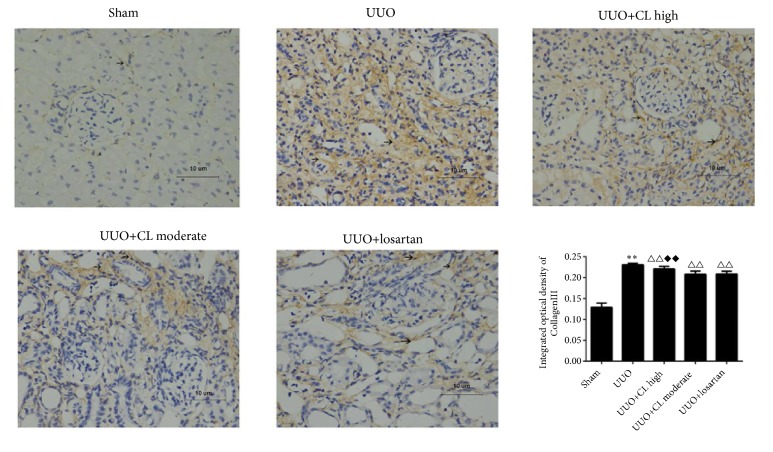
*Cili attenuates collagen deposition in kidneys after UUO*. Sixty rats were randomized to five groups: sham operation, UUO, UUO with losartan (UUO+losartan), UUO with moderate Cili dose (3 g/kg/d) (UUO+CL moderate), and UUO with high Cili dose (6 g/kg/d) (UUO+CL high). Immunohistochemistry for collagen III in kidneys and quantification. Scale bar: 10 *μ*m. N=12/group. The results are expressed as mean ± SD. *∗∗*P<0.01 vs. the sham group; △△P<0.01 vs. the UUO group; ◆◆P<0.01 vs. the losartan group.

The markers of kidney damage (BUN, serum creatinine, and 24 h urine protein) were higher in the UUO group than in the sham group (+46.9%, +84.3%, and 49.3%, all P<0.05) but were decreased after treatment with CL moderate dose (-9.8%, -12.5%, and -22.1%, resp., all P<0.01) or losartan (-11.1%, -15.3%, and -24.0%, all P<0.01) (Figure 1). Backing those results of kidney function markers, compared to the sham-operated kidneys, the obstructed kidneys from the UUO group showed tubular congestion, dilatation, and interstitial expansion (Figure 2(a)), which are pathological changes associated with kidney injury. The moderate dose of Cili freeze-dried powder (CL moderate), as well as losartan, attenuated the pathological changes induced by UUO (Figure 2(a)). In addition, collagen synthesis was high in the interstitium of kidneys from the UUO group (Figure 2(b)). The level of fibrosis was increased by UUO (+278.5%, P<0.01), but it was prevented by CL moderate dose (-16.2%, P<0.01), losartan (-22.9%, P<0.01), and resveratrol (-*∗∗∗*%, P<0.01) (Figure 2(b)). This was confirmed by immunohistochemistry for collagen III (Figure 3). Taken together, those results suggest that Cili could prevent renal fibrosis after UUO, with similar effects to those of losartan and resveratrol, which are renal protective agents.

### 3.3. Cili Powder Protects the Obstructed Kidneys from Severe Oxidative Stress

**Figure 4:**
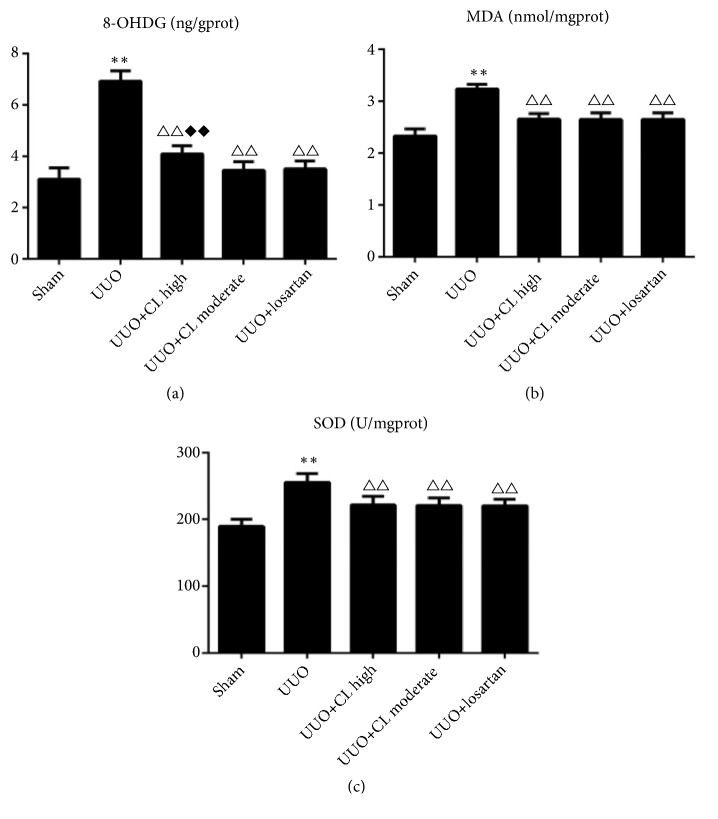
*Cili improves the markers of oxidative stress in kidneys after UUO*. Sixty rats were randomized to five groups: sham operation, UUO, UUO with losartan (UUO+losartan), UUO with moderate Cili dose (3 g/kg/d) (UUO+CL moderate), and UUO with high Cili dose (6 g/kg/d) (UUO+CL high). (a) Renal* 8-hydroxy-2*′*-deoxyguanosine* (8-OHdG) levels. (b) Renal malondialdehyde (MDA) levels. (c) Renal superoxide dismutase (SOD) levels. N=12/group. The results are expressed as mean ± SD. *∗∗*P<0.01 vs. the sham group; △△P<0.01 vs. the UUO group; ◆◆P<0.01 vs. the losartan group.

To determine the oxidative stress in the kidneys, the levels of 8-OHdG, MDA, and SOD were measured. The levels of 8-OHdG, MDA, and SOD were all increased by UUO modeling (all P<0.01) (Figure 4). Cili (both moderate and high doses) and losartan decreased the 8-OHdG, MDA, and SOD levels compared with the UUO group (all P<0.01) (Figure 4). The decrease of 8-OHdG of the UUO+CL high dose group was a little smaller than that of the UUO+losartan group (P<0.001) (Figure 4). Those results suggest that Cili prevented oxidative stress development after UUO, in a similar way as in the losartan group.

### 3.4. Cili Powder Alleviates Renal Fibrosis Which Is Associated with the Inhibition of TGF-*β*/Smads Signaling

**Figure 5:**
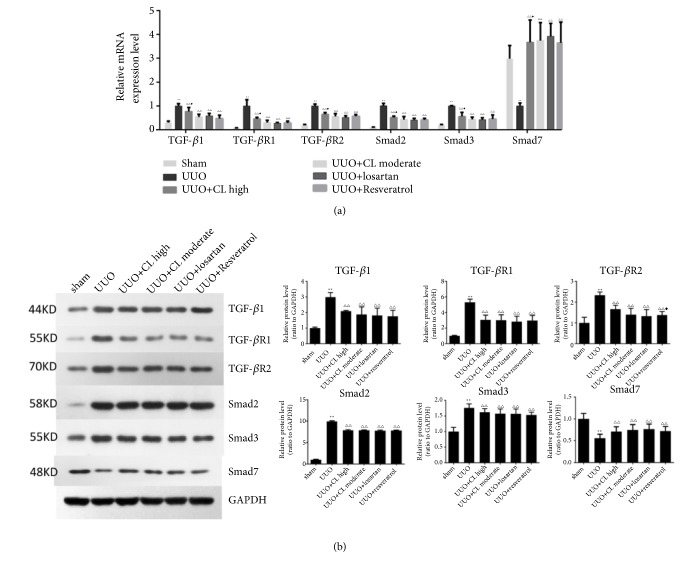
*Cili modulates the mRNA and protein expressions of proteins involved in the TGF-β/Smads pathway in kidneys after UUO*. Sixty rats were randomized to six groups: sham operation, UUO, UUO with losartan (UUO+losartan), UUO with moderate Cili dose (3 g/kg/d) (UUO+CL moderate), UUO with high Cili dose (6 g/kg/d) (UUO+CL high), and UUO with resveratrol (UUO+resveratrol). (a) Renal expression of the* TGFB1, TGFBR1, TGFBR2, SMAD2, SMAD3, and SMAD7* genes detected by quantitative RT-PCR.* GAPDH* was used as an internal control, and the relative mRNA level of each gene was normalized to the mRNA levels in the UUO group. (b) Renal expressions of the TGFB1, TGFBR1, TGFBR2, SMAD2, SMAD3, and SMAD7 proteins detected by western blot. N=12/group. The results are expressed as mean ± SD. *∗∗*P<0.01 vs. the sham group;△P<0.05 and △△P<0.01 vs. the UUO group; ◆P<0.05 vs. the losartan group.

Because the TGF-*β*/Smads pathway plays a pivotal role in renal fibrosis, the effects of Cili powder on the mRNA and protein expressions of TGF-*β*, TGF-*β* receptors, and Smads were investigated in the kidney. In the UUO group, the mRNA levels of* TGFB1, TGFBR1, TGFBR2, SMAD2, *and* SMAD3* were significantly upregulated (P<0.01), while the protective* SMAD7* was decreased (P<0.01) (Figure 5(a)). Treatment with losartan, resveratrol, or Cili powder efficiently prevented the changes in* TGFB1, TGFBR1, TGFBR2, SMAD2, SMAD3, *and* SMAD7 *(Figure 5(a)) (P<0.01 vs. UUO without treatment).

**Figure 6:**
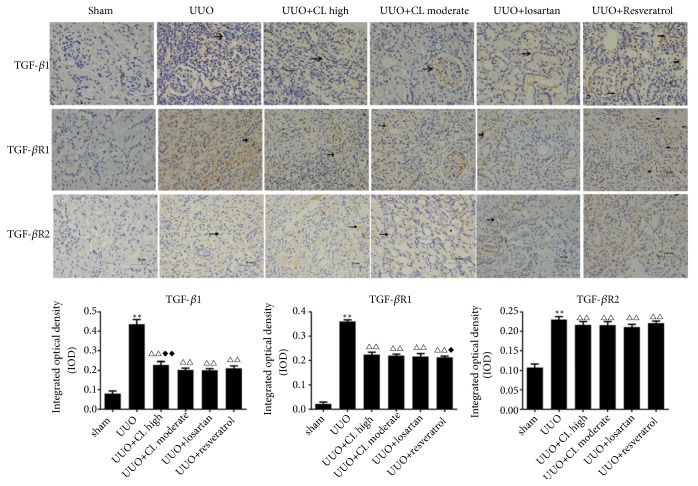
*Cili decreases the protein expression of members of the TGF-β signaling pathway in kidneys after UUO*. Representative immunohistochemistry images of key proteins in the TGF-*β* signaling in kidneys are shown with quantification. Scale bar: 10 *μ*m. Positive staining is indicated by arrows. N=12/group. The results are expressed as mean ± SD. *∗∗*P<0.01 vs. the sham group; △△P<0.01 vs. the UUO group; ◆◆P<0.01 vs. the losartan group.

**Figure 7:**
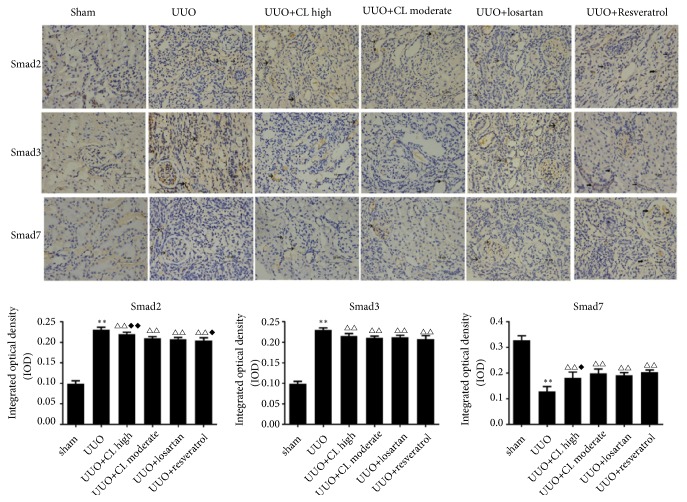
*Cili decreases the protein expression of Smad2 and Smad3 and increases the expression of Smad7 in kidneys after UUO*. Representative immunohistochemistry images of key proteins in the Smads signaling in kidneys are shown with quantification. Scale bar: 10 *μ*m. Positive staining is indicated by arrows. N=12/group. The results are expressed as mean ± SD. *∗∗*P<0.01 vs. the sham group; △△P<0.01 vs. the UUO group; ◆P<0.05 vs. the losartan group.

At the protein level, Cili moderate and high dose, losartan, and resveratrol inhibited the expression of TGF-*β*1, TGF-*β*R1, TGF-*β*R2, and Smad3, but high dose of Cili powder did not show a better antagonistic effect on the expression of any TGF-*β*/Smad proteins (Figure 5(b)). According to immunohistochemistry, Cili, losartan, and resveratrol inhibited the expression of TGF-*β*1, TGF-*β*R1, and Smad3, while maintaining Smad7 expression in obstructed kidneys (Figures 6 and 7), which is consistent with the RT-PCR results. Taken together, the results suggest that Cili powder improved renal fibrosis, possibly through the inhibition of the TGF-*β*/Smads pathway.

## 4. Discussion

Since fibrosis is involved in the pathogenesis of kidney diseases [1, 2], since Cili possesses antifibrosis properties [16, 19, 20], and since our preliminary data showed that freeze-dried Cili powder improved renal fibrosis and indexes of oxidative stress in 90 patients with stages 3-4 renal failure [24], we hypothesized that Cili could prevent the development of renal fibrosis in rat models of UUO through TGF-*β*/Smads signaling, which is known to be involved in renal fibrosis [4–6]. Therefore, this study aimed to confirm the effects of freeze-dried Cili powder on renal fibrosis and examine the TGF-*β*/Smads signaling. The results showed that Cili freeze-dried powder could effectively prevent the phenotype of renal fibrosis in UUO rat models. The beneficial effects may result from the inhibition of oxidative stress and TGF-*β*1/Smads signaling pathway. Therefore, we accepted the research hypothesis.

The incidence of chronic kidney diseases is increasing, and current treatments are limited to angiotensin II receptor blockers and angiotensin converting enzyme inhibitors. Losartan is an angiotensin II receptor inhibitor and can inhibit interstitial fibrogenesis by modulating nitric oxide synthase (NOS) isoforms and cyclooxygenase-2 (COX-2) expression [36], decreasing oxidative stress in the kidneys [37, 38] and inhibiting MCP1 and TGF-*β*1 expression in rats with UUO [39]. Resveratrol is a well-known kidney protective agent through potent reactive oxygen species scavenging abilities [40]. Resveratrol has been shown to protect against renal injury in models of diabetic nephropathy, drug-induced injury, aldosterone-induced injury, ischemia/reperfusion injury, sepsis, and UUO [40]. Therefore, losartan and resveratrol were selected as positive controls for the prevention of renal fibrosis in the UUO model.

Cili, used as a dietary supplement, has been shown to enhance the antioxidant status [17–20]. Unlike losartan, the effects of Cili extract on chronic renal diseases are still poorly understood, which limits its wide application. We previously used freeze-dried Cili powder (oral administration) to treat 90 patients with stages 3-4 chronic renal failure [24]. Therapeutic effects were achieved based on the evaluation of syndrome improvement, renal fibrosis indexes, and oxidative stress indexes. The present findings in rats confirmed the effects of* Rosa roxburghii* fruit extracts in attenuating the development of renal fibrosis in mammals. In the present study, 8-OHdG and MDA in the UUO kidneys were increased as indicatives of the renal oxidative stress induced upon injury, which could be significantly reduced by the administration of Cili freeze-dried powder. In addition, there was a higher level of SOD in the UUO kidneys as compared to the normal ones, suggesting the endogenous regulation to counteract the renal oxidative stress. Cili freeze-dried powder restored the counterregulatory rise of renal SOD in UUO rats, possibly because the antioxidant effects have been achieved. The effects observed with Cili were comparable to those of resveratrol, a potent antioxidant [40].

Notably, high-dose Cili did not result in better efficacy in preventing renal fibrosis in the UUO rat model, as revealed by the markers of kidney function, histological examination, collagen III expression, and oxidative stress. We supposed that, apart from supplying more antioxidants, high-dose Cili powder may also put a heavier excretion load on the obstructed kidney, as observed with some TCM preparations [11], limiting its efficacy at high doses, where benefits are limited by harms. A similar example is that high-dose vitamin C can induce hyperoxaluric nephropathy and progressive renal failure [41]. Therefore, since Cili is not a pure compound but a mixture of several compounds with different biological effects, a comprehensive analysis of each of the major or biologically significant Cili component will contribute to a better understanding of the effects of Cili on renal diseases.

Reactive oxygen species are important mediators of kidney damage and fibrosis, but they are not the only factors involved; many others are involved, including the various players (genes, proteins, and miRNAs) of the HIF, TGF-*β*, Notch, PKC/ERK, PI3K/Akt, NF-*κ*B, and Ang II/ROS pathways [42]. The present results provided some clues for the mechanisms underlying the effects, with a focus on the TGF*β*1/Smads pathway. The positive control resveratrol was also shown to inhibit renal fibrosis through the TGF*β*1/Smads pathway [43], supporting the present study. As the dysregulation of the TGF*β*1/Smads pathway is central in the progression of renal fibrosis [44, 45], any intervention that could modulate this pathway should play important roles in the management of the condition. Additional parameters (e.g., leukocyte infiltration [46], fibroblasts, and apoptosis [47]) need to be taken into account and to be investigated in the future. A better understanding of the mechanisms involved in renal fibrosis could lead to improved treatments in those patients.

A potential limitation of the present study was that Cili was given concomitantly with UUO. Hence, the effects observed in the present study were more preventive effects than treatment effects. Future studies should first establish renal fibrosis after UUO and then observe the effects of Cili on the kidneys. In addition, the involvement of TGF-*β*1/Smads signaling pathway will need to be confirmed using knock-out models and/or siRNAs. Furthermore, a number of other pathways are involved in renal fibrosis (e.g., HIF, TGF-*β*, Notch, PKC/ERK, PI3K/Akt, NF-*κ*B, and Ang II/ROS pathways [42]) and those pathways should be explored in order to gain a more comprehensive understanding of the effects of Cili on renal fibrosis.

## 5. Conclusions

In conclusion, the results demonstrate that Cili freeze-dried powder could effectively prevent renal fibrosis and injury in UUO rat models, suggesting the underlying mechanisms may involve the inhibition of oxidative stress and TGF-*β*1/Smads signaling.

## Figures and Tables

**Table 1:** Primers for quantitative RT-PCR.

Gene	Primer	Sequence (5′->3′)	Size (bp)
GAPDH	Forward	CATGAGAAGTATGACAACAGCCT	113
	Reverse	AGTCCTTCCACGATACCAAAGT	
SMAD2	Forward	GCCGCCCGAAGGGTAGAT	164
	Reverse	TTCTGTTCTCCACCACCTGC	
SMAD7	Forward	GGTGCGTGGTGGCATACT	144
	Reverse	GCTGACTCTTGTTGTCCGAAT	
TGFBR1	Forward	GCAATGGGCTTAGTATTCTGG	76
	Reverse	AAGGCAACTGGTAGTCTTCGTG	
TGFBR2	Forward	TGTGGAGGAAGAACGACAAGA	78
	Reverse	GAGTGAAGCCGTGGTAGGTG	
TGFB1	Forward	CATGGAGCTGGTGAAACGGAAG	74
	Reverse	GACTGGCGAGCCTTAGTTTGGAC	
SMAD3	Forward	CGATGTCCCCAGCACACAATAAC	82
	Reverse	TAGTAGGAGATGGAGCACCAAAAGG	

**Table 2:** Nutrient content of freeze-dried powder of *Rosa roxburghii.*

Item	Value	Unit
Aspartate	8.3	mg/100g
Glutamate	10	
Serine	13.1	
Glycine	88	
Threonine	11.2	
Histidine	4.4	
Alanine	58.9	
Arginine	147	
Tyrosine	7.4	
Valine	15.3	
Methionine	2	
Phenylalanine	3.6	
Isoleucine	7.6	
Leucine	3.4	
Lysine	11.5	
Proline	6.2	
All amino acids	398	
Total flavonoids	361	mg/100g
Vitamin C	6.8	mg/g
Vitamin P	0.346	
Superoxide dismutase activity	2.88 × 10^4^	U/g

## Data Availability

No data were used to support this study.
